# Effectiveness of a peanut ball device during labour on maternal and neonatal outcomes: protocol for a randomised controlled trial

**DOI:** 10.12688/f1000research.109537.2

**Published:** 2022-11-21

**Authors:** Pratibha Kamath, Muralidhar Pai, Revathi Shenoy, Sushmitha Karkada, Sonia D’souza, Judith Noronha

**Affiliations:** 1 Department of Obstetrics and Gynecological Nursing, Manipal College of Nursing, Manipal Academy of Higher Education (MAHE), Manipal, Karnataka, 576104, India; 2 Sikkim Manipal Institute of Medical Sciences(SMIMS),, Sikkim Manipal University(SMU),, Gangtok, Sikkim, India; 3 Department of Biochemistry,, Kasturba Medical College, Manipal Academy of Higher Education (MAHE), Manipal,, Karnataka, 576104, India

**Keywords:** Study protocol, Randomized controlled trial, Labour, Peanut ball, Maternal and neonatal outcome, Stress, Cortisol

## Abstract

Frequent positional changes and movements during labour is one of the recommendations by the World Health Organization (WHO) to prevent prolonged labour, thereby avoiding cesarean sections. However, labour induction, continuous fetal monitoring in supine position and immobilising the women during labour are standard practices in most private hospitals. To combat these problems and to implement WHO recommendations, the peanut ball is an effective device through which frequent positional changes will be achieved without disrupting the labour procedures. The current study aims to evaluate the effectiveness of the peanut ball device during labour on maternal and neonatal outcomes and assess the stress response induced by labour in terms of maternal and neonatal cortisol in low-risk primigravid women. The study is a prospective, block randomised controlled trial with parallel arms. A total of 768 study participants will be randomised to the peanut-ball group (intervention) and standard care group (control). The intervention group will receive different peanut ball positions during labour at or after 4 cm of cervical dilatation. The primary outcomes of the study are maternal outcome that includes measurement of duration of the active and the second stage of labour, stress level as measured by serum cortisol level at 3–4 cm and at 10 cm of cervical dilatation, mode of delivery, perception of pain, behavioural response during the active stage of labour and neonatal outcomes, which includes the pattern of fetal heart rate, APGAR score, birth injuries, and umbilical serum and salivary cortisol level. The collected data will be compared between the intervention and control groups.

**Trial Registration:** This research is registered under the CTRI (Clinical Trials Registry of India) ( CTRI/2019/08/020802) (21/8/2019).

## Abbreviations

APGAR: Appearance, Pulse, Grimace, Activity, and Respiration

CS: Cesarean Section

QACE: Qestionnaire on Assessing the Childbirth Experiences

WHO: World Health Organization

## Introduction

Childbirth is one of the happiest moments in married woman's life, and pregnancy is a special event. On the other hand, childbirth can be a very frightening experience for women, especially first births. A woman is more likely to suffer from morbidity and death during pregnancy and childbirth. Despite the fact that childbirth is a normal procedure, the majority of women who go through the labor process perceive it as a dangerous and challenging scenario. In order to effectively address these issue, it is critical to understand how these factors can affect maternal and newborn outcomes as well as to find certain nursing strategies to deal with the scenario that mediate these effects. From the start of early labor to the delivery of the baby and placenta, the birthing process takes place over the course of many hours or even a few days. The mother must progress through four phases of labor in order for the typical labor process to continue ( Hodnett *et al.*, 2013). The first phase of labor is the lengthiest. For primipara, it lasts roughly 12 to 16 hours, and for multipara, it lasts roughly 6 to 8 hours Latent, active, and transitional phases made up the first stage of labor. During the active period of labor, when contractions are stronger and more frequent, a pregnant woman is admitted to the hospital for delivery. The majority of pregnant women experience shakiness, trembling, nausea, and irritability during uterine contractions ( Al-Seady, Fadel, El-Gohary, & Marzouk, 2017).

When a woman is in labor and admitted to the hospital, the medical staff permits her to lie down so she can be restrained to the bed. Some healthcare providers only permit laboring on the bed alone and do not permit full ambulation during labor. The right of a pregnant woman to experience labor pain should be honoured ( Lagergren *et al.*, 2013) The usage of peanut balls, a non-pharmacological intervention that has been used successfully in some countries, is one of several complementary treatments and non-pharmacological interventions that are used during labor to lessen discomfort and shorten the time it takes.

## Background

Even after the strong recommendations of the World Health Organization regarding mobility and upright positions to prevent delay in the first stage of labor, increase in induction during labor, and continuous fetal monitoring ( Calik *et al.*, 2018) made position changes challenging during labor. Even if a mother is constrained to bed, frequent changes of maternal positions that facilitate pelvic movements should be given during labor ( Lawrence *et al.*, 2013). The absence of changes in the position of the mother during labor can lead to prolonged labor and increases the chance of cesarean birth due to failure to progress or descend ( Zwelling, 2010; Akyıldız *et al.*, 2021). The peanut ball is considered an effective intervention to overcome this obstacle and promote the different positions during labor.

The peanut ball is an alternative to the conventional birth ball. The birthing ball is preferably used during the pregnancy period ( Ghasab *et al.*, 2019; Mirzakhani *et al.*, 2015), whereas the effectiveness of the peanut ball is elicited even during labor ( Outland & Alvarado, 2019). The peanut ball is a curved peanut-shaped ball that has a middle indentation that allows the women to place the ball between and below the knees for allowing either the lateral, supine or sitting position, which increases pelvic dimensions, promotes progressive fetal rotation and descent during second-stage labor and ultimately promote the progress of labor ( Zwelling, 2010; Roth *et al.*, 2016). Several studies have shown a reduction in either the length of labor ( Roth *et al.*, 2016; Tussey CM, 2015) or a reduction in the cesarean birth ( Outland & Alvarado, 2019) with use of the peanut ball in immobile women.

In 2015, Tussey *et al.* reported in a randomised controlled trial that effective utilisation of peanut ball during labor in the epidural woman has shortened the second stage of labor by 11 minutes and reached clinical significance in decreasing the length of time of the second stage of labor by 29 minutes. In addition, only 10% of those women who delivered with a peanut ball had a caesareans birth compared to 21% in the control group (p < .05) ( Tussey CM, 2015).

Roth and his colleagues in the year 2016 demonstrated in a randomised controlled study that the first stage of labor was significantly reduced for the nulliparous women (p = .018), but not for the multiparous women after the use of peanut ball. But no statistically significant difference was found in the length of the pushing stage for either nulliparous or multiparous women ( Roth *et al.*, 2016). These findings were supported by the quasi-experimental study done by Hickey & Savage, and they found out that peanut ball use during labor substantially (50%) decreased the rate of cesarean sections among women (164) laboring with epidurals ( Hickey & Savage, 2019) However, these studies’ results are not statistically significant to prove that the peanut ball alone has decreased the labor duration ( Outland & Alvarado, 2019; Roth *et al.*, 2016; Tussey CM, 2015; Hickey & Savage, 2019).

Furthermore, Ahmadpour and team concluded in their systematic review and meta-analysis that the 645 women who used the peanut ball during labor have no statistically significant difference between the two groups in cesarean section rate and the length of the first stage of labor. Therefore, the study recommended conducting more rigorous randomised controlled trials to evaluate the effectiveness of the peanut ball during labor ( Ahmadpour *et al.*, 2021).

Even the Grenwik, *et al.*, study in 2019 supported the statement in their systematic review and summarised that more research is needed to prove the statistical significance in decreasing labor duration after the peanut ball use ( Grenwik *et al.*, 2019).

In the Indian research literature, there have been no randomised studies conducted on the peanut ball interventions ( Jayasudha *et al.*, 2021). That is why we, the researchers, through the randomised clinical trial, aimed at evaluating the effectiveness of a peanut ball device during labor on maternal and neonatal outcomes and assessing the stress response induced by labor in terms of maternal and fetal cortisol in low-risk primigravid women. If found to be effective, the proposed large-scale clinical trial, the peanut ball intervention can be implemented routinely in maternal healthcare settings for safe and comfortable delivery, which can ultimately prevent prolongation of labor and decrease the proportion of postpartum hemorrhage and stress level among the mother.

## Research objectives

The following objectives are formulated:

1. To determine the effectiveness of the use of a peanut ball device during labor in terms of following maternal outcomes:
  • Duration of the active stage of labor
  • Duration of the second stage of labor
  • Nature of delivery
  • Need for pain medications
  • Perception of pain
  • An occurrence of postpartum haemorrhage
2. To determine the effectiveness of the use of a peanut ball device during labor in terms of following neonatal outcomes.
  • Fetal heart rate patterns
  • Neonatal Intensive Care Unit (NICU) admissions
  • APGAR score (Appearance (skin colour), Pulse (heart rate), Grimace (reflex irritability), Activity (muscle tone) score and Respiration) at the time of birth at one minute and five minutes.
  • Birth injuries
3. To identify and compare the behavioural response, stress level, childbirth experience and maternal satisfaction among low-risk primigravid women during the active stage of labor between the experimental and control group.
4. To identify and compare the stress level of neonates between the intervention and control.

## Hypotheses

The trial is designed to test the hypotheses at a 0.05 level of significance. The following hypotheses were formulated.

H1: There will be a significant difference in the maternal and neonatal outcomes during labor between the intervention and control group.

H2: There will be a significant difference in the behavioural response score between the intervention and control group.

H3: There will be a significant difference in the maternal cortisol levels between the intervention and control group.

H4: There will be a significant difference in the neonatal cortisol levels between the intervention and control group.

H5: There will be a significant difference between the childbirth experience score between the intervention and control group.

## Methods

### Study design

A prospective block randomised controlled trial with parallel groups is the research design adopted for the study. Block randomisation of 64 blocks with a block size of 12 each is generated using a Research randomiser, an online random number generator by a study statistician. A senior nurse who is working in the labor ward and who is not directly involved in the study will be generating the sequence. Concealment allocation will be achieved using a sequentially numbered opaque and sealed envelope (SNOSE), where participants will be assigned randomly either to receive the peanut ball intervention or standard care.

**Figure 1.**
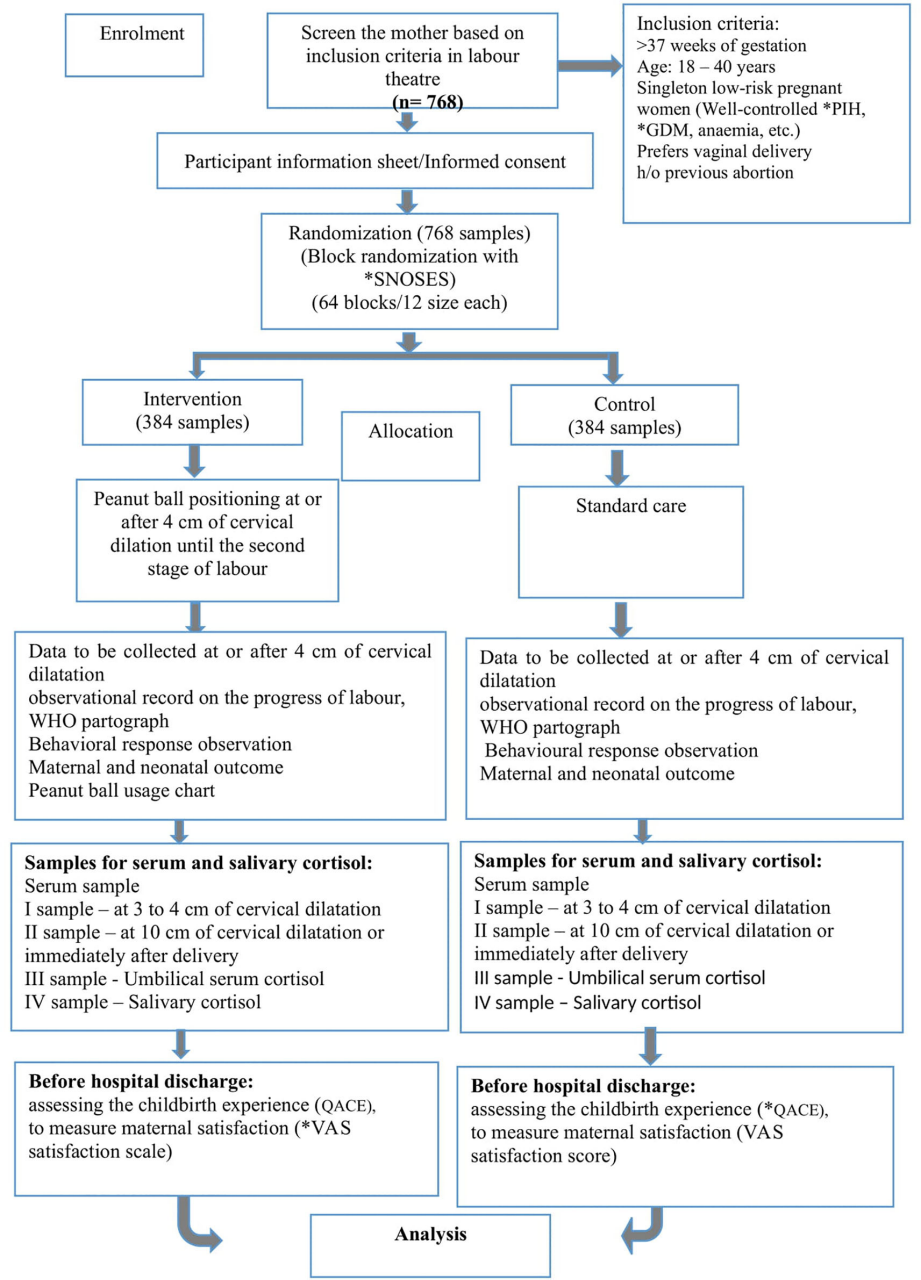
Consolidated standards of reporting clinical trial (CONSORT) flow chart. *PIH: Pregnancy Induced Hypertension, *GDM: Gestational Diabetes Mellitus, *VAS: Visual Anologue scale, WHO: World Health Organization. *SNOSEs: Sequentially Numbered Opaque and Sealed Envelopes, *QACE: Questionnaire on Assessing the Childbirth Experiences.

All study materials can be found as *Extended data* ( Kamath, 2022a– f). The study will adhere to the Consolidated Standards of Reporting Trials (CONSORT) guidelines ( Figure 1) ( Sally Hopewell, 2008) and the protocol follows the Standard Protocol Items: Recommendations for Interventional Trials (SPIRIT) guidelines ( Kamath, 2022g).

### Study setting and population

This study will be conducted in labor theatre of selected tertiary care hospital from Udupi District, Karnataka, India. Udupi district is hailed as one of the most progressive districts in the state about women and their empowerment. The percentage of women receiving antenatal care in the district according to 2011 census is 79.76% and share of institutional deliveries are 99.76%. Total bed strength of the selected tertiary care Hospital is 2400. Obstetrical and Gynecological (OBG) department is a multi-specialty department providing high-quality patient care and consists of a well- qualified team of Obstetricians and Gynecologists divided into 5 units and are dedicated to providing excellence. OBG department itself has a total of 250 beds with trained staff nurses with 1:1 staff patient ratio in Labor wards, to take care of any adverse effects at the time of intervention. Labor ward has 20 beds with well-equipped which contains Fetal heart rate monitoring machine (NST Machine) and tocography to record the uterine contractions. Population for the study is all low risk primigravid antenatal women admitted in labor theatre for labor or elective labor induction. An estimated 768 low-risk primigravid women will be recruited. This research is approved by Manipal Academy of Higher Education, Institutional review board (Reg no. ECR/146/Inst/KA/2013/RR-16).

### Eligibility criteria

The eligibility criteria include low-risk primigravid women who are aged between 18 to 40 years, singleton pregnancy and in cephalic presentation, gestational age of 37.0 to 41.6 weeks, well-controlled pre-eclampsia, and gestational diabetes mellitus, history of abortions, well-controlled/moderate anaemia, history of mild intrauterine growth restriction (IUGR), willing to use the peanut ball during the first stage of labor and preferring a vaginal delivery.

### Exclusion criteria

The exclusion criteria include women who are known to have any major fetal anomalies and very low and late preterm, a high-risk antenatal mother with any pregnancy-related complications like severe pre-eclampsia/eclampsia, preterm labor, placenta praevia, malpresentation during the study, contraindication for vaginal delivery, multigravida, multiple pregnancies, had any sort of fracture of leg/any major problem in the past and having weight more than 90 kg.

### Sample-size

The expected variations in the research outcome variable determines the sample size for the study; n=2Z1−α2+Z1−β2σ2/d2 (where n is the sample size per group, Z1−α2 = 1.96 at α = 0.05, $Z_{1-\beta }$ = 0.84 (power at 80% power), σ = standard deviation of the primary outcome variable stress level of the mother as measured by serum cortisol level = 2.58 μg/dl ( Stjernholm, Nyberg, Cardell, & Höybye, 2016), d = clinically significant difference = 0.55 μg/dl. The sample size, calculated with an anticipatory attrition rate of 10%, is 768.

### Recruitment and consent

All the low-risk primigravid women at or after 37 weeks of gestation attending the outpatient services at tertiary care hospitals for an antenatal check-up will be oriented about the implementation of peanut ball during labor by using the peanut ball positions chart prepared by premier birth tools and by showing the video on peanut ball during labor prepared by the researcher.

Thereafter, the mothers will be screened in the labor room based on the inclusion criteria. The research team will explain the research purpose and procedure to the eligible mothers and, if they are willing to participate, informed written consent will be taken.

### Participant allocation/randomisation

Subsequently, the low risk primigravid women at 2–3 cm of cervical dilatation preferring a vaginal delivery will be block randomised to peanut ball intervention group or standard care control group by an external member (labor room nurse) with the help of SNOSEs. The research assistant and research nurse carrying out data collection, data entry, and data analysis will be blinded to group allocation.

### Intervention

**Figure 2.**
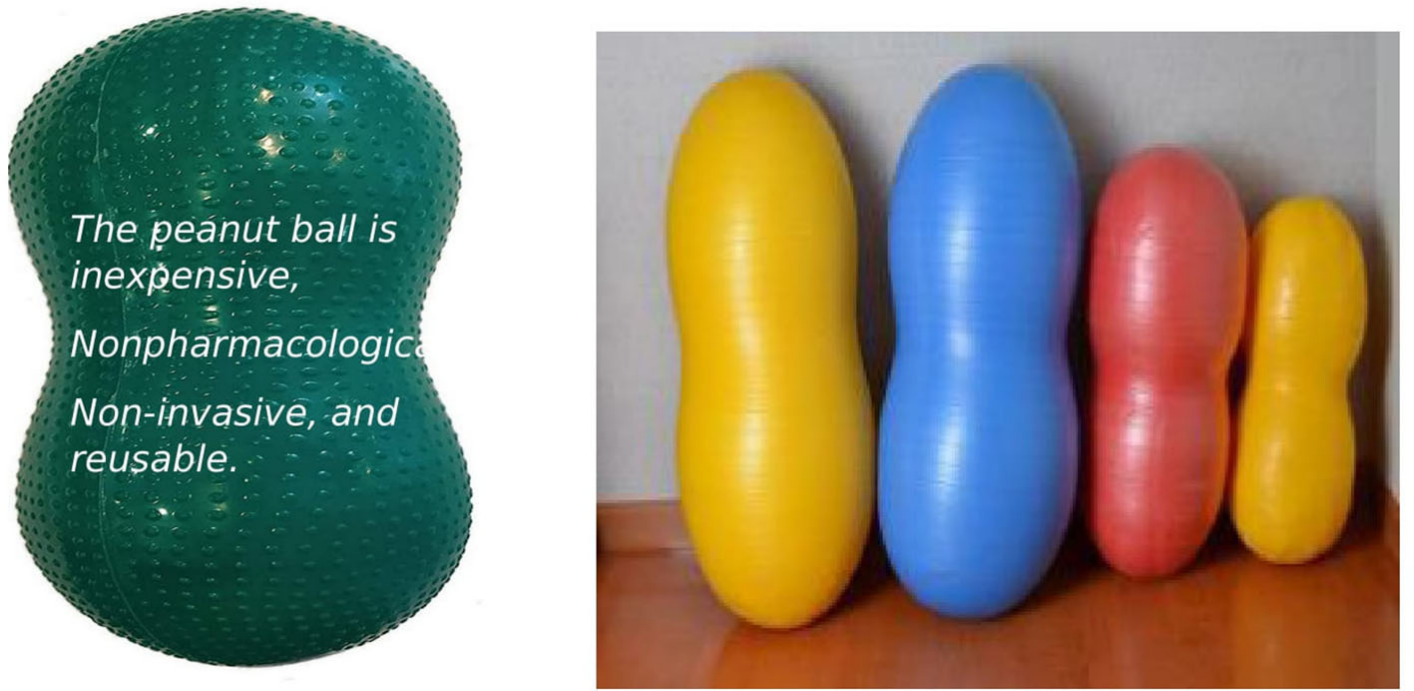
Different sizes of peanut balls.

The peanut ball positioning is done at or after 4 cm of dilatation by a trained nurse-midwife whenever the mother is lying or sitting on the bed. Peanut balls are available in four sizes: 40 cm, 50 cm, 60 cm, and 70 cm to fit different participants as reported by Grant & Clutter in 2014. The researcher will make sure that the suitable size of the ball is given to the woman as recommended by the premier birth tools as per the height of the mother, that is: 40 cm for women whose height is under 5′3″, 50 cm is the most common size and exclusivly used for women with 5′3″ to 5′ 6″ height, 60 cm for women 5′7″ or taller or overweight women, and 70 cm used only to sit on and straddle on the peanut ball as reported by Grant C in 2015. All the sizes of peanut balls will be available in the labor room ( Figure 2) to use during labor and will be covered with clean peanut ball covers. A suitable peanut ball will be provided to the mother to give four main peanut ball positions: sitting, semi-sitting, side-lying, and tuck position until the mother is ready to push during the second stage of labor.

Adherence to intervention protocols and procedures will be done continuously by research assistants throughout the intervention by being with the patient and educating and comforting the mother.

### Standard care

The control group of women will receive the standard/routine care provided in the labor room of the tertiary care setting.

### Criteria for discontinuing from the study

Participants will be recruited only after informed written consent. Participants participation in this study is voluntary and they can withdraw their participation at any time, without giving any reasons. They will still get the usual routine care during labor, and non-participation will not have any negative consequences on their subsequent medical treatment or relationship with the treating obstetrician and midwife. If the participant withdraws from the study before data collection is completed, their data collected till the withdrawal will be used in the study report.

### Outcome measures

Maternal and neonatal outcomes will be measured by the research assistants at different points of time and compared between the intervention and control groups. The maternal outcome includes measurement of the duration of the active stage of labor, duration/pushing effort in the second stage of labor, mode of delivery, the need of pain medications, perception of pain, behavioural response during the active stage of labor, serum analysis of cortisol level, the proportion of postpartum hemorrhage, and neonatal outcome includes a pattern of fetal heart rate, APGAR score (Apgar V, 1958), birth injuries, and salivary cortisol level.

During the active stage of labor blood samples will be collected twice from the mothers in both groups for serum cortisol analysis. The 1 ^st^ sample will be collected when cervical dilatation is 3–4 cm and the 2 ^nd^ sample will be collected after full dilatation of the cervix and these will be sent to lab for analysis. An umbilical cord blood sample will be collected from the newborn at the time of birth to check the serum cortisol level, and salivary cortisol will be collected from the newborn within one hour of birth to check the salivary cortisol level. An observational record on the progress of the labor will be done for both the groups once they reach 4 cm of cervical dilatation by using the WHO partograph Both the groups will be observed for their behavioral responses for any two hours during the first stage of the labor with the cervical dilatation ranging from 4–7 cms by using the behavioral response observational checklist (Karkada *et al.*, 2010) Both the neonatal and maternal outcomes will be recorded by using the outcome of the labor assessment tool ( Kamath, 2022f) and WHO Partograph, and during postnatal period before getting discharged from the hospital, ie., 3–4 days after the delivery, women from both of the groups will be assessed for their childbirth experience by using a questionnaire for assessing the childbirth experience (QACE) tool ( Carquillat, P. *et al.*, 2017). Maternal satisfaction regarding assistance provided during labor/delivery will be measured by using VAS (visual analogue scale) patient satisfaction score ( Hayes and Patterson, 1921).

### Outcome of the labor assessment tool

The researcher prepared a record of the outcome of labor to document maternal and neonatal outcomes. The maternal outcome consists of 18 items, such as duration of labor, nature of delivery, need of pain medications, objective assessment of pain, evaluation of maternal and fetal cortisol levels and postpartum hemorrhage etc. The neonatal tool includes details of APGAR score at birth, weight of the newborn, neonatal injuries during birth and particulars of NICU admission etc. ( Kamath, 2022f).

### Serum cortisol and neonatal cortisol assessment

Blood samples will be collected twice during labor to determine the stress level. The first blood sample will be collected when cervical dilatation is 4–5 cm, and the second will be collected when cervical dilatation is of 10 cm from both groups and these will be sent to the biochemistry lab for analysis. After complete coagulation, the blood sample will be centrifuged, and then the serum will be separated for analysis of cortisol concentration. A blood sample from the umbilical cord will be taken at the time of birth to check the serum cortisol level of the baby, and saliva will be collected within one hour of birth by using swab stick by keeping it in baby’s mouth for two minutes to check the salivary cortisol level. The serum will be separated by centrifuging the cord blood. Using the principles of electrochemiluminescence immunoassays, cortisol levels will be measured in serum and cord blood using the Cobas -602 e-immunoassay analyser. Assay reagents are obtained from Roche Diagnostics. The saliva samples will be immediately frozen at 20° C until analysis. The free cortisol will be determined by ELISA.

### Behavioral response observation checklist

This observation checklist, prepared by Karkada EC *et al*., is used in this study by obtaining permission from the author to assess the behavioural responses of low-risk primigravid women during the first stage of labor. A behavioural response checklist has three areas of observation, i.e., the behavioural response in between contractions which has 20 items, facial response during a contraction, which contains eight items; behavioural responses during contractions, which includes 20 items; and lastly, physiological parameters, which have two items. Overall, the tool includes 50 items. The study encompasses negative and positive responses ( Karkada, Noronha, & Dsouza, 2010).

### Questionnaire for assessing the childbirth experience, vaginal birth, or cesarean (QACE)

QACE questionnaire is self reporting tool screens for any negative experiences during child birth. It consists of six main thematic categories. These thematic categories are expectations (three items), sensory experiences (two items), perceived control (six items), relationship with caregivers and the midwife (four items), emotions (seven items), and the first moments with the baby (three items). Additional ten and the other three items are to measure causal indicators that potentially influence the childbirth experience. The response format is a 4-point Likert scale, ranging from “Totally”, “In part”, “Not so much”, and “Not at all” ( Carquillat, P. *et al.*, 2017).

### VAS patient satisfaction scale

The researchers modified the VAS into a patient satisfaction scale to assess the satisfaction level of low-risk primigravid women during labor. It is a 10 cm scale with smileys indicating expression of satisfaction, with the scoring: 0 – indicates no satisfaction and score ranging between 1–3 – mild level of satisfaction, 4–7 – moderate level of satisfaction, and 8–10 – high level of satisfaction ( Hayes and Patterson, 1921).

### Validity and reliability of the study tools

The validity (item Content Validity Index (I-CVI) and Scale Content Validity Index (S-CVI/Ave) and reliability ( Table 1) of the study tools were established by giving the tools, along with the criteria checklist, to a panel of nine experts from different fields, such as the women and child health department of physiotherapy, department of statistics, department of Obstetrics and Gynecological Nursing, and experts from OBG department, Kasturba Hospital Manipal to review the study tools. Content validity is established in terms of relevancy, accuracy, and appropriateness. The item Content Validity Index (I-CVI) and Scale Content Validity Index (S-CVI/Ave) of the tools were calculated based on recommendations by Polit and Beck (2012).

**Table 1.** Results of the testing of the instruments. *I-CVI- Item - content validity index, *S-CVI- Scale level - content validity index.

Instruments	Content validity		Reliability	
	Mean *I-CVI	*S-CVI/Ave	Aspect of reliability	Reliability score
Structured observational record on the outcome of the labor	1	1	Internal consistency	There was absolutely 100% agreement between two raters: r=1
Behavioural response observational checklist	1	1	Internal consistency	Intra class coefficient agreement. r=0.97
Questionnaire for assessing the childbirth experience vaginal birth or caesarean (QACE)	1	1	Internal consistency	Intra class correlation coefficient: r=0.88
VAS satisfaction scale to measure maternal satisfaction	1	1	Stability	Coefficients of stability: r=0.89

### Data entry and storage

The research assistant will do coding and data entry. The primary investigator will review the data entered for any discrepancies such as data entry errors, missing values etc. One of the co-authors will check the data entry errors by randomly selecting data sheets. A separate laptop will be used for the project store data and can be accessed only by the research assistant and primary investigator.

### Data analysis

Statistical analysis will be performed using SPSS software version 16. The data analysis will be done based on the objectives and hypotheses of the study. We will follow the intention to treat analysis (ITT). The sample characteristics are measured using descriptive statistics. Two independent samples t-test/Mann-Whitney U-test (based on normality of data) will be used to determine the effectiveness of the usage of peanut ball on the maternal outcomes of labor. Associations among the variables will be computed using the Chi-square test (for categorical variables) and two independent samples t-test/Mann-Whitney U-test or one-way ANOVA/Kruskal Wallis test (for continuous variables). A comparison of behavioural response and stress level of low-risk primigravid women among experimental and control groups will be done using repeated measures of ANOVA. A comparison of childbirth experiences will be done using an independent t-test.

### Monitoring

Data monitoring committee will be formed, which includes methodology experts, subject experts and statistician.

### Ethics and dissemination

Approval has been obtained from Head of the Unit - Obstetrical and Gynecological department and Medical Superintendent Kasturba Hospital Manipal for this study. Ethical clearance for the study was obtained from the Kasturba Medical College and Kasturba Hospital, Institutional Ethics committee Manipal (Reg No: ECR/146/1nst/KA/2013/RR-16). The trial is registered under the Clinical Trial Registry of India (CTRI/2019/08/020802). A written participant information sheet ( Kamath, 2022b) will be given regarding the details of the study, and it will be explained to all low-risk primigravid women before enrolment to the study. Their involvement benefits and harm (none) as well as whom to contact in case of doubt will be explained to the participants. Written informed consent ( Kamath, 2022c) from the participants will be obtained before involving them in study.

### Confidentiality

Confidentiality of the research data collected will be maintained strictly as per the ethical standards. Only the research assistants and the researchers will have access to the participants’ data in the study. The data and results from this study may be presented at conferences and published in scientific journals without revealing the identity of the participants.

### Injury compensation

During the study period, if any medical problem arises as a direct result of the study intervention, the researchers will be responsible for ensuring proper medical care is provided to the participant. Suppose a participant suffers from any injury (physical/mental) or disease/illness as result of the correctly implemented study procedures. In that case, the researcher is responsible for that unless the injury or disease relates to the mothers negligent or reckless act.

### Dissemination plans

The time of sharing data is within one year after completion of the trial. The findings will be disseminated to participants, healthcare professionals, the public, and other relevant groups by attending scientific conferences and publishing in reputed journals.

## Discussion

Labor is a complex phenomenon in which the passenger (fetus) goes through the passage (pelvis) using the power (contractions) to deliver a healthy neonate ( Michelle & Gayle, 2020). In order to accelerate the labor a series of interventional strategies have been adopted, and one among them is use of peanut ball during labor. Utilisation of peanut ball has proven effective in reducing cesarean section rates, drop in the length of the first stage of labor by more than 90 minutes and the second stage was reduced by 22.3 minutes. Additionally, the need for vacuum extraction and forceps delivery is decreased in the group using the Peanut Ball. It also use of peanut ball helps in labor progress by easing labor pain and reducing the discomfort of contractions, which indirectly lowers anxiety and increases a mother's positive childbirth experiences ( Tussey CM, 2015). The patient satisfaction was also noted in a qualitative study, which is done among 200 mothers, the study found out that, 118 laboring women who used peanut ball during their labor gave positive feedback in support of use of the Peanut Ball. Positive comments included that the Peanut Ball provided comfort, facilitated the progress of labor, and helped with the position. And 71 % would recommend the use of the Peanut Ball to other mothers ( Payton, 2015).

Globally, the peanut balls are extensively utilised by midwives to deliver the women through vaginal mode, but in India it is underutilised. Jayasudha, *et al*. in the year 2021, did the quasi-experimental study in India, in which peanut ball use during the first stage of labor is explored ( Jayasudha *et al.*, 2021). The study lacks a controlled setting; the stakeholders consider methodologically rigorous research evidence to make a decision related to policymaking. However, there is currently no proof that the peanut ball, used as a birthing ball, has any effect on women. The potential benefits of employing the peanut ball in India must be further investigated, especially given the current high rate of caesarean sections.

In the present study, a clinical trial with block randomisation to investigate the effectiveness of peanut ball in improving maternal and neonatal outcomes is planned in the Indian context to make the appropriate clinical decision. The effect of the peanut ball for labor among low-risk primigravid women in India will be the subject of a first-ever study.

For the study the participant recruitment began in the month February 2020. To date, we have enrolled and randomised 550 low-risk primigravid mothers. Out of the total, 225 mothers were recruited to the intervention and 225 mothers were recruited for the control group. Mothers’ unacceptance to utilise the peanut ball during labor is a challenge faced during the initial part of the study. To encounter this, all the primigravid women at or after 37 weeks of gestation attending antenatal outpatient units were given informational material about the “Peanut ball use during labor”. This educational material has oriented and familiarised the mothers with peanut ball and its uses during labor which ultimately increased the rate of peanut ball acceptance and use during labor.

Along with that, coronavirus disease 2019 (COVID-19) imposed a lockdown, and after that, institutional restrictions to resume research activities to mitigate new cases of infection had made the study come to a halt. Recruitment for the study was stopped, and we lost participants for around five months. Due to this situation, we have fallen behind the recruitment timeline. To combat this, we utilised this duration to initiate a systematic review that will bring new evidence to implement peanut ball during labor.

Besides these challenges, there are two possible limitations in this study, first, ascertainment bias, as the investigators are aware of the intervention the participants are receiving. In this project, the investigator and participants will know the allocation group for the women, as the woman will either utilise the peanut-ball or not, so they cannot be blinded. The strategy implemented to reduce the ascertainment bias is allocation concealment using SNOSEs by the labor room senior nurse on duty. The research assistant and research nurse carrying out data collection, data entry, and data analysis will be blinded to group allocation. The second limitation is compliance with the intervention during labor. To tackle this, a trained nurse will be present throughout the labor process and encourage and motivates the mothers to use the peanut ball continuously.

Despite these possible limitations, this novel approach will be a promising intervention to promote labor progress and prevent unnecessary caesarean section and instrumental birth during the second stage of labor.

## Conclusion

In this paper, researchers described the study design and the methodological approach adopted to evaluate the effectiveness of use of peanut ball during labor. Expected results of the study are usage of peanut ball helps in widening the pelvic outlet thereby supporting the natural progression of birth by increased maternal-fetal circulation, decreased pain and labor stress, and fewer abnormal fetal heart rate patterns, decreased duration of labor, less risk for postpartum hemorrhage and reduce the number of cesarean section rate. This project would thus enable to reduce maternal morbidity and mortality and can be used as a guide to implement peanut ball use in the labor rooms of tertiary health care settings in the Indian context and all over the world.

## Data availability

### Underlying data

No underlying data is associated with this article.

### Extended data

This project contains following extended data:

Figshare: Demographic proforma.docx. https://doi.org/10.6084/m9.figshare.19122233 ( Kamath, 2022a)

Figshare: Participant Information Sheet.docx. https://doi.org/10.6084/m9.figshare.19122230 ( Kamath, 2022b)

Figshare: 18 Informed Consent English.doc https://doi.org/10.6084/m9.figshare.19122242 ( Kamath, 2022c)

Data are available under the terms of the Creative Commons Zero “No rights reserved” data waiver (CC0 1.0 Public domain dedication).

Figshare: Screening Tool.docx. https://doi.org/10.6084/m9.figshare.19122239 ( Kamath, 2022d)

Data are available under the terms of the Creative Commons Attribution 4.0 International license (CC-BY 4.0).

Figshare: Tool III modified partograph.docx. https://doi.org/10.6084/m9.figshare.19122236 ( Kamath, 2022e)

Data are available under the terms of the Creative Commons Zero “No rights reserved” data waiver (CC0 1.0 Public domain dedication).

Figshare: Structured observational record on outcome of the labor.docx https://doi.org/10.6084/m9.figshare.19122227 ( Kamath, 2022f)

Data are available under the terms of the Creative Commons Attribution 4.0 International license (CC-BY 4.0).

## Reporting guidelines

Repository: SPIRIT checklist for ‘Effectiveness of a peanut ball device during labor on maternal and neonatal outcomes: protocol for a randomised controlled trial’. https://figshare.com/s/5e8db2fc8dee7a7e06e5 ( Kamath, 2022g)

Data are available under the terms of the Creative Commons Attribution 4.0 International license (CC-BY 4.0).
