# Supplementary material for: Effectiveness of a peanut ball device during labour on maternal and neonatal outcomes: protocol for a randomised controlled trial
Source: F1000Res. 2022 Nov 21;11:717. Originally published 2022 Jun 29. [Version 2] doi: 10.12688/f1000research.109537.2 (PMC9727314; doi:10.12688/f1000research.109537.2)
Supplement: Peer review history

# Peer review: Reviewer response for version 2

**DOI:** 10.5256/f1000research.141098.r156236

**Published:** 2022-12-06

**Authors:** Vidya Seshan

**Affiliations:** 1 Maternal and Child Health Department, College of Nursing, Sultan Qaboos University, Muscat, Oman

Greetings, 

 Authors have responded to my comments very well.

 I have no more comments. Hence approving the manuscript at the present status.

 Thank you 

 Best regards 

 Dr. Vidya Seshan

Is the study design appropriate for the research question?

Yes

Is the rationale for, and objectives of, the study clearly described?

Yes

Are sufficient details of the methods provided to allow replication by others?

Yes

Are the datasets clearly presented in a useable and accessible format?

Yes

Reviewer Expertise:

women's Health ,Maternal Health, teaching stratergies

I confirm that I have read this submission and believe that I have an appropriate level of expertise to confirm that it is of an acceptable scientific standard.

# Peer review: Reviewer response for version 1

**DOI:** 10.5256/f1000research.121047.r153356

**Published:** 2022-10-27

**Authors:** Sabitha Nayak

**Affiliations:** 1 Department of OBG Nursing, Nitte Usha Institute of Nursing Sciences, NITTE (Deemed to be University), Mangaluru, Karnataka, India

This is an interventional/RCT study where the investigators could find out the effectiveness of peanut ball device during labour on maternal and neonatal outcomes is a novel study in Indian settings. The study protocol seems good enough with clear cut objectives and appropriate methodology as mentioned. The tools used are also appropriate. There seems to be no harm on the mother or the fetus. The study results may be of help to generalise as the sample size is large. It can be implemented as a clinical practice by all the health professionals mainly midwives and obstetricians working in the labour unit.

Is the study design appropriate for the research question?

Yes

Is the rationale for, and objectives of, the study clearly described?

Yes

Are sufficient details of the methods provided to allow replication by others?

Yes

Are the datasets clearly presented in a useable and accessible format?

Yes

Reviewer Expertise:

NA

I confirm that I have read this submission and believe that I have an appropriate level of expertise to confirm that it is of an acceptable scientific standard.

# Response

**Published:** 2022-10-28

**Authors:** Pratibha Kamath

**Affiliations:** Manipal Academy of Higher Education, Manipal, India

Dear Madam,

 Thank you so much for reviewing.

 With Warm Regards

 Pratibha

 PI of the project

# Peer review: Reviewer response for version 1

**DOI:** 10.5256/f1000research.121047.r150084

**Published:** 2022-10-17

**Authors:** Vidya Seshan

**Affiliations:** 1 Maternal and Child Health Department, College of Nursing, Sultan Qaboos University, Muscat, Oman

Title: Effectiveness of a peanut ball device during labor on maternal and neonatal outcomes: protocol for a randomized controlled trial

 This is a study to see the effect of peanut ball devices during labor. It’s a proposal written by the researcher to the journal. Well-structured methodology.

 Needs some minor revisions:

- The reference style is not followed consistently in the introduction.
- The introduction is more of a literature review than an explanation related to the study. The researcher has quoted the funding under study setting and population; this is not required to be in this section, it should be written at the end related to funding.
- The setting is not explained well. Only one sentence about the settings. Needs more explanation of the study setting for the readers to understand. Eligibility criteria and exclusion criteria need a separate heading. The discussion and conclusion are very shallow.

Is the study design appropriate for the research question?

Yes

Is the rationale for, and objectives of, the study clearly described?

Yes

Are sufficient details of the methods provided to allow replication by others?

Yes

Are the datasets clearly presented in a useable and accessible format?

Yes

Reviewer Expertise:

women's Health ,Maternal Health, teaching stratergies

I confirm that I have read this submission and believe that I have an appropriate level of expertise to confirm that it is of an acceptable scientific standard, however I have significant reservations, as outlined above.

# Response

**Published:** 2022-10-28

**Authors:** Pratibha Kamath

**Affiliations:** Manipal Academy of Higher Education, Manipal, India

Dear Madam,

 Thank you very much for reviewing the article and for your suggestions, I will incorporate and upload again.

 With Warm Regards

 Pratibha

 PI of the project
